# Switching From a 6° to a 20° Valgus Prosthetic Trochlear Groove Improved the Forgotten Joint and Oxford Knee Scores After Kinematically Aligned Total Knee Arthroplasty

**DOI:** 10.1016/j.artd.2025.101930

**Published:** 2025-12-26

**Authors:** Stephen M. Howell, Ahmed Zabiba, Alexander J. Nedopil, Maury L. Hull

**Affiliations:** a Department of Biomedical Engineering, University of California, Davis, CA, USA; b University of California at Davis School of Medicine, Sacramento, CA, USA; c Orthopädische Klinik König-Ludwig-Haus, Lehrstuhl für Orthopädie der Universität Würzburg, Würzburg, Germany; d Department of Orthopedic Surgery, University of California, Davis, CA, USA; e Department of Mechanical Engineering, University of California, Davis, CA, USA

**Keywords:** Kinematic alignment, Total knee arthroplasty, Forgotten joint score, Oxford knee score, KOOS

## Abstract

**Background:**

In kinematically aligned (KA) total knee arthroplasty (TKA) using a femoral component with the traditional 6° valgus prosthetic trochlear groove (PTG), patients reported a lower Forgotten Joint Score (FJS) when the quadriceps line of pull was laterally misaligned to the groove, with an incidence of 89%. It remains unclear whether switching to a KA-optimized femoral component with a 20° valgus PTG, which properly aligns the quadriceps line of pull, can improve the FJS and the Oxford Knee Score (OKS).

**Methods:**

The analysis of single-surgeon series of KA TKAs included 145 cases with a KA-optimized 20° valgus PTG and 292 cases with a 6° valgus PTG. Each participant reported their FJS and OKS at 2 years and underwent a postoperative coronal long-leg scan.

**Results:**

The 20° group had a 6-point higher FJS (79) and a 16% lower incidence of poor FJS (<40) (8%) compared to the 6° group. Additionally, 73% and 22% achieved an excellent (48-42) or good (41-34) OKS, compared to 64% and 20% with a 6° valgus PTG. The FJS in the 20° group was 7 and 20 points higher in the coronal plane alignment of the knee (CPAK) types 2 and 3.

**Conclusions:**

Surgeons performing KA TKA should consider switching to a KA-optimized femoral component with a 20° valgus PTG, as this option improves the FJS and OKS, lowers the risk of a poor FJS, and is especially useful for CPAK 2 and 3, with no apparent disadvantages in CPAK 1, 4, and 5.

**Level of Evidence:**

III.

## Introduction

Despite advancements in alignment techniques and implant design for total knee arthroplasty (TKA), some patients still report lower-than-expected patient-reported outcome (PRO) scores. In kinematically aligned (KA) TKA, it is hypothesized that modifying the prosthetic trochlea of the femoral component may enhance the Forgotten Joint Score (FJS) and the Oxford Knee Score (OKS) [[1], [2], [3], [4], [5], [6]].

**Figure 1:**
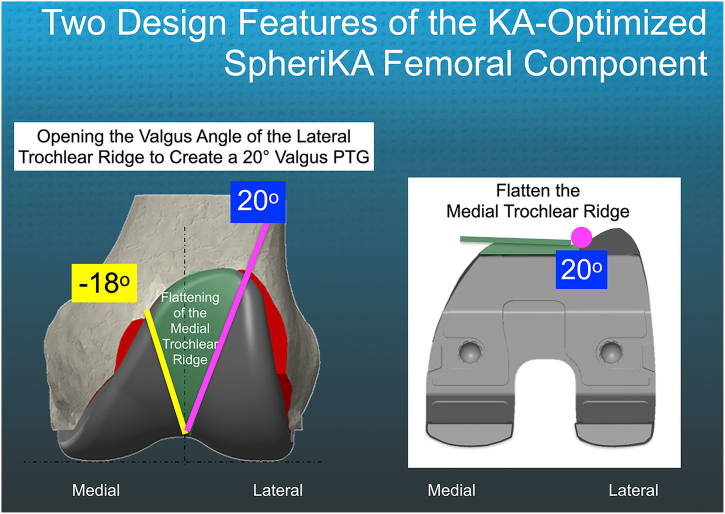
The KA-optimized femoral component features a trochlea with the lateral ridge opened to create a 20° valgus PTG and a flattened medial ridge (dark green), which accommodates the −18° varus to 20° valgus variability in the anterior arthritic trochlear groove that occurs in 99% of patients [7].

**Figure 2:**
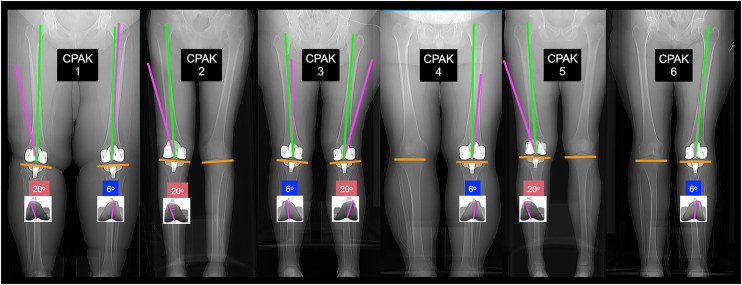
Postoperative scanograms of types 1-6 CPAK show normal alignment of the QLOP (green line) within or medial to the PTG (magenta line), and abnormal lateral misalignment to the PTG that lowers the FJS [1,3,6]. The QLOP is normally aligned at the 20° valgus PTG and is laterally misaligned to the 6° valgus PTG in CPAK 3 and 6.

To create a KA-optimized femoral component, 2 design changes are proposed for the traditional mechanically aligned (MA) femoral component, which features a traditional 6° valgus prosthetic trochlea (PTG). The first change involves widening the opening of the lateral trochlear ridge from 6° to 20° valgus to reduce the risk of lateral misalignment of the quadriceps’ line of pull (QLOP) to the PTG [6] (Fig. 1). Lateral misalignment of the QLOP is common with a 6° valgus PTG, especially in the coronal plane alignment of the knee (CPAK) types 1, 2, 3, and 6. This misalignment can lower the FJS compared to cases where the QLOP is correctly oriented within or medial to the groove [3,5] (Fig. 2).

The second change involves flattening the medial trochlear ridge to accommodate the variability of the anterior arthritic trochlear groove, which in 99% of cases varies between −18° varus and 20° valgus [7] (Fig. 1). Flattening the medial trochlear ridge reduces the risk of overstuffing the anterior femoral compartment, which can happen when the anterior arthritic trochlear groove overlaps the medial prosthetic trochlear ridge [1].

**Figure 3:**
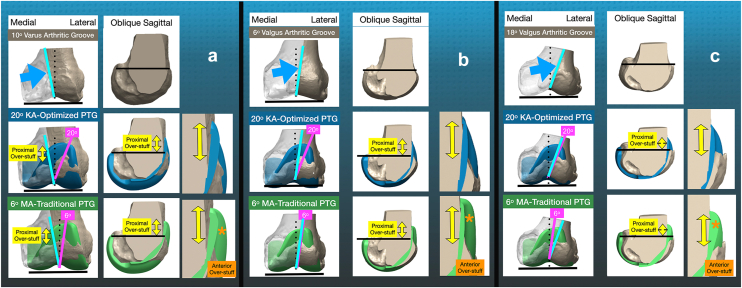
Schematics illustrate the different effects of overstuffing the femoral part of the anterior compartment caused by a KA-optimized femoral component with a 20° valgus PTG (blue) and a MA-femoral component with a 6° valgus PTG (green). Both lead to proximal overstuffing (yellow double-headed arrows) above the upper border of the native trochlea (black line), which is reported to average 17 mm in another design regardless of whether the native groove (cyan line) is 10° varus (a), 6° valgus (b), or 18° valgus (c) relative to the distal femoral joint line [8]. However, the 6° valgus PTG causes greater anterior overstuffing proximal to the native groove than the 20° version because its lateral ridge is more medial (orange asterisk). MA, mechanically aligned.

Widening the lateral trochlear ridge from 6° to 20° valgus and flattening the medial trochlear ridge are necessary to counteract overstuffing caused by the prosthetic trochlea extending proximal to the native trochlea by an average of 17 mm (range, 7 to 32 mm) [8] (Fig. 3). This explains why understuffing >2 mm relative to the native trochlear peaks is preferred in KA, as it results in better FJS and OKS than peak restoration [1].

The present study analyzed KA TKAs performed with either a 20° or 6° valgus PTG. The focus was on evaluating the 2-year FJS and OKS. The study tested 2 hypotheses: (1) The 20° valgus PTG will yield higher FJS and OKS scores and a lower percentage of patients with a poor FJS (defined as less than 40) compared to the 6° design and (2) The 20° valgus PTG will enhance the FJS in specific CPAK types without negatively impacting the scores in other types. By identifying the most effective trochlear design for all knees and for specific CPAK types, this research aims to assist surgeons in making better-informed decisions when performing KA TKA.

## Material and methods

### Patient selection

The study involved patients treated by a single surgeon who underwent primary KA TKA for osteoarthritis, with Kellgren–Lawrence Grades III to IV, using a femoral component with either a 20° or 6° valgus PTG starting in November 2021. All patients treated in 2019 had a 6° valgus PTG (GMK SpheriKA or Sphere, Medacta International, Castel San Pietro, Switzerland, www.medacta.com, accessed November 5, 2023). In November 2021, the 20° KA-optimized femoral component, which has the same mediolateral and anteroposterior (AP) dimensions as the 6° component, making them interchangeable, with the only distinguishing feature being the trochlear morphology, was added to the hospital inventory as a continuous update. The surgeon reviewed a list of 4 or 5 patients scheduled for KA TKA. The 20° component was always used whenever the mediolateral and AP dimensions of the patient’s femur size matched an available 20° version, regardless of limb alignment, knee deformity, location of compartmental arthritis, patient age, sex, or body mass index. All primary TKAs were performed with KA using manual instruments, regardless of the severity of varus or valgus deformity or flexion contracture, a standard since 2009. To undergo surgical treatment, each patient met the Centers for Medicare & Medicaid Services guidelines for medical necessity for TKA.

The process of including and excluding patients in the study is as follows: patients with a history of septic knee arthritis, avascular necrosis, previous fractures in the limb, hip, or ankle arthroplasty, or lower-extremity neurologic disorders were excluded after reviewing their charts. Patients were included if they had completed an FJS (scoring 100 as the best and zero as the worst) and an OKS (scoring 48 as the best and zero as the worst) with at least 2 years of follow-up, regardless of whether they had previous TKA on the opposite knee or bilateral primary KA TKA. Those with a postoperative anterior–posterior long-leg scanogram free of malrotation were included in the CPAK analysis.

### Outcome measures

On the day of the initial consultation, each patient provided demographic information and completed the OKS, and the Knee Injury and Osteoarthritis Outcome Score Joint Replacement (100 is best, zero is worst) on an iPad. Additionally, the physician assistant recorded knee extension, flexion, and alignment deformity using a long-arm goniometer.

### KA-optimized implant design

The KA-optimized femoral component features a lateral ridge opening that creates a 20° valgus trochlear groove and a flattened medial ridge, as shown in Figure 1 [1]. The insert includes a medial socket for ball-in-socket articulation and a lateral flat surface that mimics the morphology of the native knee [9]. Additionally, a posterior recess in the insert allows for retention of the posterior cruciate ligament (PCL), which helps restore native internal tibial rotation [10]. These features increase the FJS by an average of 16 and 10 points, respectively, compared with PCL-retaining and PCL-substituting implants after KA TKA [11,12].

The KA-optimized tibial component features an asymmetric axial footprint to ensure maximum coverage of the tibial resection. When the largest size is positioned correctly within the cortical boundary of the tibia, the AP axis of the insert aligns parallel to the flexion–extension plane of the native knee following KA principles [13]. These features are crucial for restoring native knee kinematics [10,[14], [15], [16], [17]].

### Surgical technique

The surgeon used a mid-vastus approach and manual instruments to perform the KA TKA with PCL retention. Caliper measurements of the femoral bone resections confirmed that the femoral restores the prearthritic articular surface with a reported accuracy of within 0 ± 0.5 mm, which is essential for optimizing postoperative FJS and OKS [18]. This accuracy surpasses that of robotics, and the learning curve is negligible for inexperienced surgeons [[19], [20], [21], [22]]. The sagittal position of the femoral component was set with a 9 mm intraosseous positioning rod, which accurately determines the flexion of the femoral component, with an average flexion and variability (± standard deviation (SD)) of 1° ± 2° relative to the anatomic axis [23].

Three surgical steps were performed to ensure the knee was balanced and the correct insert thickness was chosen. A balanced TKA is defined by restoring native forces in the medial and lateral tibial compartments and ligament laxities [24,25]. The first step is to fine-tune the varus–valgus alignment of the tibial resection until a tight, rectangular extension gap is achieved during a varus-valgus laxity test relative to the distal femoral resection when a spacer block is interposed. The second step is to set the tibial component within ±2° of the prearthritic slope, which the surgeon accomplishes by inserting an angel wing through the medial side of the saw slot on the tibial resection guide and adjusting the flexion–extension until it contacts both the anterior and posterior rims of the articular surface of the medial tibial plateau, which has a negligible risk of requiring a recut [26]. The third step involves using an insert goniometer to select the optimal insert thickness by identifying the thickness that offers the greatest internal tibial rotation at 90° of flexion, without causing a loss of knee extension or anterior lift-off from the baseplate. Choosing the correct insert thickness is crucial because using an insert 2 mm thicker doubles the forces in the medial and lateral tibial compartments, causes a 3° loss of extension, and results in 3 mm of posterior and paradoxical translation of the femoral component, which can lead to knee stiffness [27,28].

The patella was resurfaced using a freehand resection technique, which creates a symmetric resection, in contrast to the use of cutting guides [29,30]. A broad fan-shaped saw initiated the osteotomy at the inferior pole of the patella, just posterior to the insertion of the patellar tendon. It was carried proximally, behind the insertion of the quadriceps tendon. A 10 mm-thick, anatomically shaped patellar component was implanted. This freehand resection technique allows for the thinnest possible patella remnant while preserving the tendinous attachments. In 90% of TKAs, the thickness of the patella-implant construct is within ±1 mm of the intraoperative patella thickness [31]. Those who prefer the caliper resection technique should be aware that it is less accurate than the freehand flush technique, as the resurfaced patella is either thicker (up to 12 mm) or thinner (up to 7 mm) than the intraoperative patella in thickness in 44% and 28% of TKAs, respectively [32]. No patient underwent a lateral release.

### Postoperative care and follow-up

Before discharge, each patient underwent an AP, non–weight-bearing, long-leg scanogram of both legs, performed using a computer tomographic scanner with a radiation dose of 0.5 mSv, which is lower than that of a long-leg radiograph [33,34]. Patients received an electronic questionnaire at 24 months to complete the FJS and OKS, and to report any complications and revision surgery.

### Radiographic analysis

The following alignment variables measured on the computer tomographic scanogram include the hip–knee–ankle angle, distal lateral femoral angle, and proximal medial tibial angle, as shown in Table 1 [33]. Using these measurements, each patient was assigned to a CPAK type [5,35].

**Table 1:** The table summarizes the patient’s preoperative characteristics and PRO scores, and the postoperative radiographic long-leg alignment for patients with a 20° and 6° valgus PTG.

Pre-operative characteristics	20° valgus PTG	6° valgus PTG	Significance
Number of KA TKAs	145	292	
Age	68 ± 8 y	68 ± 8 y	NS, *P* = .4789
Sex	80 females, 65 males	162 females, 130 males	NS, *P* = .9515
Body mass index	30 ± 6 kg/m^2^	30 ± 6 kg/m^2^	NS, *P* = .7713
Knee extension	8 ± 7^o^	7 ± 6^o^	NS, *P* = .1065
Knee flexion	119 ± 9^o^	113 ± 7^o^	*P* = < 0.0001
Preoperative PRO Measures			
OKS (48 is best, 0 is worst)	25 ± 8 points	23 ± 8 points	*P* = .0246
KOOS JR	51 ± 13 points	49 ± 14 points	NS, *P* = .2835
Postoperative Radiographic Alignment			
Hip–knee–ankle angle	180^o^ ± 3^o^ (168^o^ varus to 189^o^ valgus)	179^o^ ± 3^o^ (170^o^ varus to 187^o^ valgus)	NS, *P* = .7528
Distal lateral femoral angle	87^o^ valgus ± 3^o^ (80^o^ varus to 95^o^ valgus)	87^o^ valgus ± 2^o^ (79^o^ varus to 93^o^ valgus)	NS, *P* = .7103
Proximal medial tibial angle	86^o^ varus ± 2^o^ (80^o^ varus to 92^o^ valgus)	86^o^ varus ± 2^o^ (79^o^ varus to 93^o^ valgus)	NS, *P* = .8612

Reported as mean ± SD.

KOOS JR, Knee Injury and Osteoarthritis Outcome Score Joint Replacement.

### Ethical considerations

Advarra CIRIBA, an institutional review board, provided an exempt determination (Pro00087373) for a retrospective analysis of deidentified patient data obtained from a prospectively archived records database.

### Statistical analysis

A sample size calculation was conducted for the FJS and OKS using a Wilcoxon–Mann–Whitney test calculator (G-Power v3.1.9.6, https://gpower.macupdate.com/, accessed May 24, 2025). Employing a 2-sided test instead of the more appropriate one-sided test resulted in a more conservative sample size and addressed a reviewer’s comment. The significance level was set at 0.05, and the power (1 - Beta) was set at 90%. The effect size (d) was 0.5, based on a minimally clinically important difference and SDs of 14 and 28 points for the FJS [36], and 5 and 10 points for the OKS [37]. Consequently, the minimum sample size per group was 110 patients for both the FJS and OKS.

Statistical software was used to calculate the mean and SD for dependent variables that followed a normal distribution, and the median and interquartile range for those that did not (JMP Pro, version 18.0.1, http://www.jmp.com, accessed December 29, 2024). Each OKS was classified as excellent (48 to 42), good (41 to 34), fair (33 to 27), or poor (<27) based on Kalairajah's criteria [38]. A Wilcoxon Two-Sample Test was employed to assess the significance of differences in FJS and OKS scores between patients with 20° or 6° valgus PTG. Additionally, a Pearson's chi-square test was performed to evaluate differences in the proportions of patients with poor FJS (<40) and those with excellent and good Kalairajah OKS classifications between the 2 patient groups. For each CPAK type, the Wilcoxon Two-Sample Test was used to assess differences in FJS and OKS between patients with 20° or 6° valgus PTG.

## Results

The analysis included 145 patients with a 20° valgus PTG and an average follow-up of 25 ± 2 months, and 292 patients with a 6° valgus PTG and an average follow-up of 29 ± 6 months. Table 1 summarizes the preoperative demographics, extension and flexion, and PROs, as well as postoperative radiographic findings, showing no clinically significant differences between those with a 20° and 6° valgus PTG.

**Figure 4:**
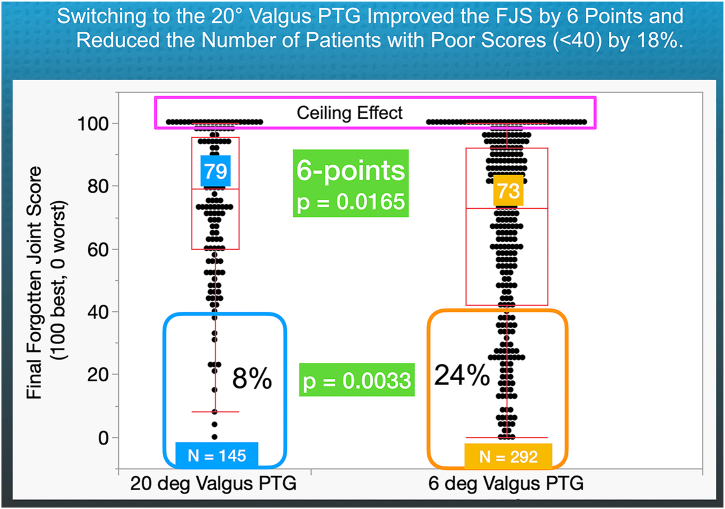
The quantile box plots show that for the 20° valgus PTG, the median FJS was 6 points higher (*P* = .0165), and the percentage of patients with a poor FJS (less than 40) was 16% lower than for those with a 6° valgus PTG (*P* = .0033). This is notable because the ceiling effect (magenta rectangle) reduces the FJS's ability to detect differences.

**Figure 5:**
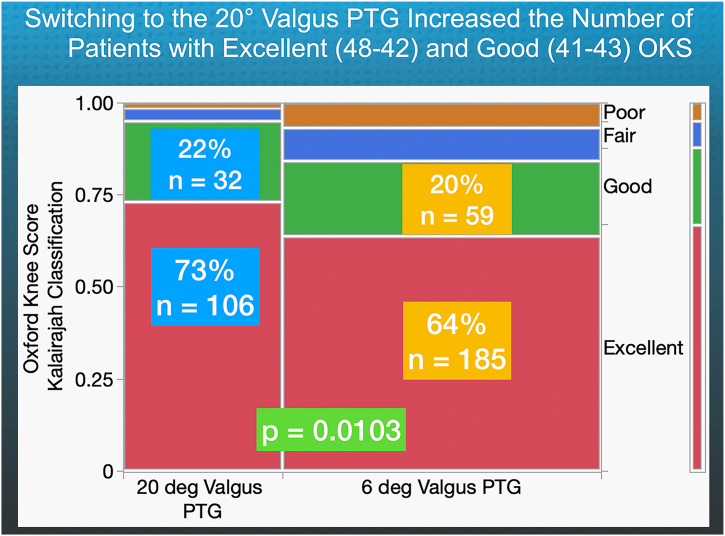
The contingency table shows that 73% and 22% with the 20° valgus PTG had an excellent (48-42) and good (41-34) OKS, which was significantly higher than the 64% and 20% values for those with the 6° valgus PTG (*P* = .0103).

In all patients, the median FJS for the 20° KA-optimized femoral component was 79, which is higher than the 73 observed for the 6° valgus PTG, with a significant difference (*P* = .0165) (Fig. 4). The 8% of patients with a poor FJS (<40) in the 20° valgus PTG group was significantly lower than the 24% in the 6° valgus PTG group (*P* = .0033). Furthermore, 73% of patients rated their OKS as excellent or good, compared to 64% of those with the 6° PTG; this difference was also statistically significant (*P* = .0103) (Fig. 5).

**Figure 6:**
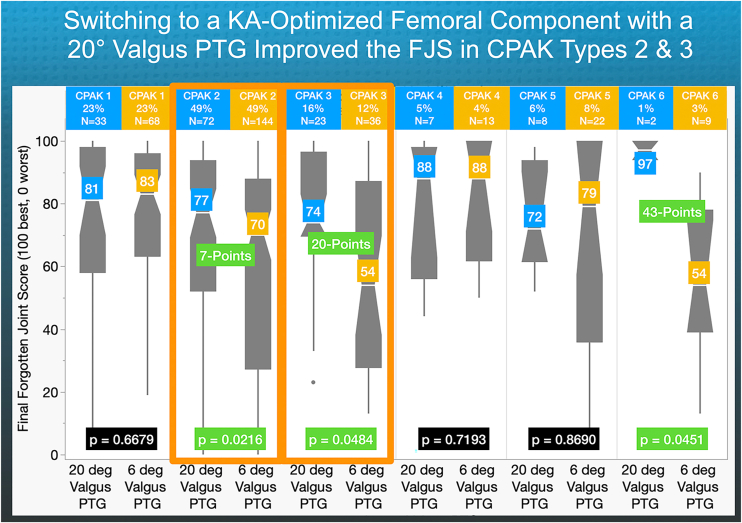
For each CPAK type, the quantile box plots show that the median FJS with a 20° valgus PTG was 7 and 20 points higher in CPAK 2 and 3, respectively, than with a 6° valgus PTG.

The CPAK type analysis revealed that the median FJS for the 20° PTG was 7 and 20 points higher in CPAK types 2 and 3, respectively, when compared to the 6° PTG (*P* = .0216 and *P* = .0484) (Fig. 6). Additionally, the 20° valgus PTG did not adversely affect the FJS in CPAK types 1, 4, 5, and 6. However, the sample sizes for CPAK types 4, 5, and 6 were too small for a meaningful analysis.

**Figure 7:**
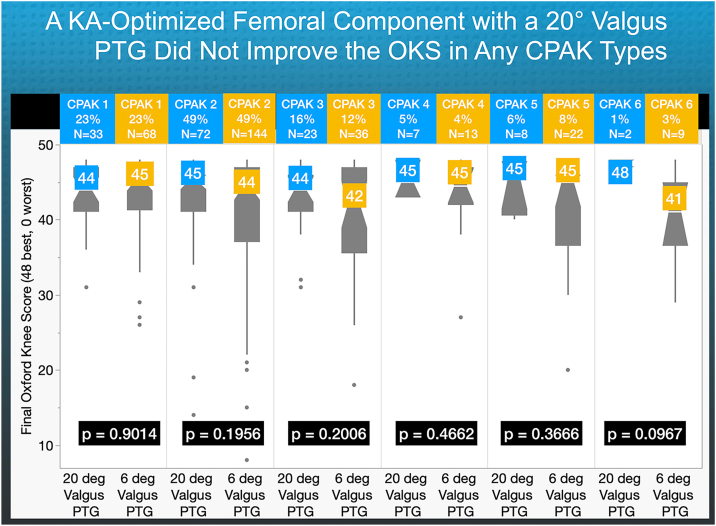
For each CPAK type, there were no significant differences in the OKS between the 20° and 6° valgus PTG (*P*≥ = 0.0967).

Lastly, the CPAK type analysis showed that the median OKS for both the 20° and 6° valgus PTG were not significantly different, which may be attributed to a ceiling effect (*P* = .0967) (Fig. 7).

## Discussion

The most significant findings of this study on KA TKA, which compared 2-year FJS and OKS between patients treated with a femoral component featuring a KA-optimized femoral component with a 20° valgus PTG and the traditional 6° valgus PTG, were that (1) the 20° valgus PTG yielded better FJS and OKS scores and a lower percentage of patients with a poor FJS (<40) compared to the 6° design and (2) the 20° valgus PTG provided better FJS in CPAK types 2 and 3 without negatively impacting the scores in types 1, 4, and 5. These findings may assist surgeons in selecting a femoral component during KA TKA.

Although the 20° valgus PTG increased the overall FJS by 6 points, it is important to interpret this difference, as the FJS has a minimal clinically important difference of 14 points [28]. The overall 6-point higher FJS score should not be ignored, as its ability to discriminate is limited in this study due to a ceiling effect, with participants reaching the maximum score of 100—17% of subjects with the 20° valgus PTG and 14% with the 6° valgus PTG. Additionally, the KA-optimized femoral component led to 16% fewer patients with a poor FJS (<40).

The KA-optimized femoral component has significant potential to improve FJS in patients with valgus limbs and those with distal apex joint-line obliquity. This component helps decrease the risk of lateral misalignment of the QLOP, which occurs in 35%, 89%, and 50% of cases classified as CPAK types 2, 3, and 6, respectively [5,6]. Strong evidence suggests that the KA-optimized femoral component should be considered for CPAK 2 and 3, as it can improve FJS by 7 and 20 points, respectively, compared to traditional components with a 6° valgus PTG. However, the relevance of using a 20° valgus PTG in CPAK 4, 5, and 6—making up only 5%, 6%, and 1% of patients—could not be assessed due to the small numbers in each group. Furthermore, there is no apparent drawback to using a 20° valgus PTG in CPAK 1, 4, and 5, as evidence indicates that switching from a 6° valgus PTG did not lower the FJS.

Surgeons performing KA TKA, particularly those hesitant to treat knees with valgus deformities, should find these results reassuring. Prior studies indicate that KA TKAs with a 6° valgus PTG restored to their valgus prearthritic phenotype had a higher rate of reoperation for anterior knee pain or patellofemoral instability and worse FJS compared to those with postoperative varus phenotypes [3,5,39]. A recent radiographic analysis suggests that these adverse outcomes should not be attributed to the integrity of the medial collateral ligament, as it is rarely elongated in osteoarthritic knees with valgus deformity [40].

The KA-optimized femoral component design offers a significant clinical advantage by typically under-stuffing the peaks of the medial and lateral trochlear ridges by more than 2 mm. This design yields comparable or even better FJS, OKS, and Knee Injury and Osteoarthritis Outcome Score Joint Replacement when compared to peak restoration within ±2 mm [1]. Understuffing of the medial and lateral peaks with the prosthetic trochlea compensates for the anterior compartment overstuffing caused by the extension of the prosthetic trochlea proximal to the native trochlea, which averages 17 mm (with a range of 7 to 32 mm) [8]. Surgeons should avoid the temptation to anteriorize the posteromedial articular surface by even 1 mm to restore the medial peak height. Doing so could result in a decrease of 40 points in the FJS and 14 points in the OKS [18].

### Limitations

It is crucial to recognize several limitations of this study. First, all procedures were performed by a single experienced surgeon who specializes in KA TKA. This may limit the generalizability of the results to other surgeons with varying levels of experience. However, research suggests that the learning curve for performing KA with manual instruments is minimal [19]. Both experienced and novice surgeons achieve similar levels of accuracy concerning distal and posterior femoral resections, as measured by the deviation from the target thickness using a caliper. This accuracy surpasses that achieved with robotic instrumentation [16,17,19]. Therefore, the findings of this study may still be applicable regardless of the surgeon's experience level.

Second, the power analysis was designed to ensure a conservative sample size. One aspect involved setting the power level to 0.90 instead of the more common 0.80. Additionally, a 2-tailed test was used rather than a 1-tailed test, despite the hypothesis being 1-sided—that the FJS and OKS would be higher for those with 20° valgus PTG than for those with 6° valgus PTG—which indicated a minimum sample size of 90 patients per group. Since our study included 145 patients with a 20° valgus PTG and 292 with a 6° valgus PTG, it was overpowered rather than underpowered.

Third, the sagittal orientation of the femoral component did not influence the FJS and OKS results in this study because the same alignment method—using a 9 mm intraosseous positioning rod to set the sagittal orientation—was used for both the 20° and 6° versions. The technique is highly accurate, with an average flexion of 1° relative to the anatomic axis and high precision, as evidenced by a SD of ±2° [20].

Fourth, the 6° difference in preoperative knee flexion between the 20° and 6° groups should not impact the FJS and OKS results, as systematic analyses have shown that preoperative range of motion has an inconsistent effect on postoperative clinical outcomes after TKA, despite having a direct correlation with postoperative motion [41].

Fifth, the analysis of the patellar tilt angle was not performed. Therefore, excluding a Merchant view analysis from our study.

Finally, the classification of patella height into patella alta and baja was not performed. However, there is little reason to suspect that the frequency of each would differ between the 20° and 6° groups, as both cohorts received the same surgical instruments and perioperative care. Additionally, their preoperative demographics (except for preoperative flexion), PROs, and postoperative limb and component alignment were comparable (Table 1).

## Conclusions

The KA-optimized femoral component with a 20° valgus PTG has the potential to become the standard for KA TKA, as it improves the FJS and OKS and reduces the risk of a poor FJS score (<40 points) compared to traditional designs with a 6° valgus PTG. It is especially suitable for CPAK types 2 and 3. Additionally, it can be considered an alternative to the 6° valgus PTG for CPAK types 1, 4, and 5, as it shows no detectable disadvantages.

## CRediT authorship contribution statement

**Stephen M. Howell:** Writing – review & editing, Writing – original draft, Visualization, Validation, Supervision, Software, Resources, Project administration, Methodology, Investigation, Funding acquisition, Formal analysis, Data curation, Conceptualization. **Ahmed Zabiba:** Writing – review & editing, Writing – original draft, Visualization, Validation, Supervision, Software, Resources, Project administration, Methodology, Investigation, Funding acquisition, Formal analysis, Data curation, Conceptualization. **Alexander J. Nedopil:** Writing – review & editing, Writing – original draft, Visualization, Validation, Supervision, Software, Resources, Project administration, Methodology, Investigation, Funding acquisition, Formal analysis, Data curation, Conceptualization. **Maury L. Hull:** Writing – review & editing, Writing – original draft, Visualization, Validation, Supervision, Software, Resources, Project administration, Methodology, Investigation, Funding acquisition, Formal analysis, Data curation, Conceptualization.

## Conflicts of interest

Stephen M. Howell receives consulting fees and royalties, is on the speakers' bureau of, receives research support as a principal investigator from Medacta International (Castel San Pietro, Switzerland, www.medacta.com) and receives royalties, financial or material support from Elsevier. Alexander J. Nedopil is a paid consultant for Medacta, Think Surgical, and Smith & Nephew; and is on the medical/orthopaedic publications editorial/governing board of KSSTA.

Maury L. Hull receives research support from and is a paid speaker for Medacta International (Castel San Pietro, Switzerland, www.medacta.com) and is on the medical/orthopaedic publications editorial/governing board of Journal of Biomechanics; Knee Surgery, Sports Traumatology, Arthroscopy; Bioengineering.

The other author declares no potential conflicts of interest.

For full disclosure statements refer to https://doi.org/10.1016/j.artd.2025.101930.

## Appendix A Supplementary data

Conflict of Interest Statement for Howell

Conflict of Interest Statement for Nedopil

Conflict of Interest Statement for Zabiba

Conflict of Interest Statement for Hull
