# Supplementary material for: Switching From a 6° to a 20° Valgus Prosthetic Trochlear Groove Improved the Forgotten Joint and Oxford Knee Scores After Kinematically Aligned Total Knee Arthroplasty
Source: Arthroplast Today. 2025 Dec 26;37:101930. doi: 10.1016/j.artd.2025.101930 (PMC12796931; doi:10.1016/j.artd.2025.101930)
Supplement: Conflict of Interest Statement for Hull [file mmc4.docx]

# INDIVIDUAL CONFLICT OF INTEREST STATEMENT

***American Association of Hip and Knee Surgeons***

(Adopted from the American Academy of Orthopaedic Surgeons disclosure statement)

The following form **must be filled out completely and submitted by each author (example, 6 authors, 6 forms).**

**All items require a response. If there is no relevant disclosure for a given item, enter "*None*.”**

**Manuscript Title:** Switching from a 6° to a 20° Valgus Prosthetic Trochlear Groove Improved the Forgotten Joint and Oxford Knee Scores after Kinematically Aligned Total Knee Arthroplasty

1. Royalties from a company or supplier (The following conflicts were disclosed)

- None

2. Speakers bureau/paid presentations for a company or supplier (The following conflicts were disclosed)

Paid speaker by Medacta USA, Inc.

3A. Paid employee for a company or supplier (The following conflicts were disclosed)

- None

3B. Paid consultant for a company or supplier (The following conflicts were disclosed)

- None

3C. Unpaid consultants for a company or supplier (The following conflicts were disclosed)

- None

4. Stock or stock options in a company or supplier (The following conflicts were disclosed)

- None

5. Research support from a company or supplier as a Principal Investigator (The following conflicts were disclosed)

- Medacta USA, Inc.

6. Other financial or material support from a company or supplier (The following conflicts were disclosed)

- None

7. Royalties, financial or material support from publishers (The following conflicts were disclosed)

- None

8. Medical/Orthopaedic publications editorial/governing board (The following conflicts were disclosed)

Journal of Biomechanics; Knee Surgery, Sports Traumatology, Arthroscopy; Bioengineering

9. Board member/committee appointments for a society (The following conflicts were disclosed)

- None

**Each author must sign AND print or type his/her name, date and submit a separate form**

In addition, one BLINDED Conflict of Interest form (no author names used) should be submitted per manuscript with all author disclosures.

Maury L. Hull


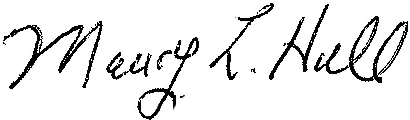
Author Name (Print or Type) Maury L. Hull Author Signature Date 02/18/25
